# On the Use of the Pearson Correlation Coefficient for Model Evaluation in Genome-Wide Prediction

**DOI:** 10.3389/fgene.2019.00899

**Published:** 2019-09-26

**Authors:** Patrik Waldmann

**Affiliations:** Department of Animal Breeding and Genetics, The Swedish Universiy of Agricultural Sciences, SLU, Uppsala, Sweden

**Keywords:** genomic selection, model comparison, accuracy, bias–variance trade-off, coefficient of determination

## Abstract

The large number of markers in genome-wide prediction demands the use of methods with regularization and model comparison based on some hold-out test prediction error measure. In quantitative genetics, it is common practice to calculate the Pearson correlation coefficient (*r^2^*) as a standardized measure of the predictive accuracy of a model. Based on arguments from the bias–variance trade-off theory in statistical learning, we show that shrinkage of the regression coefficients (i.e., QTL effects) reduces the prediction mean squared error (MSE) by introducing model bias compared with the ordinary least squares method. We also show that the LASSO and the adaptive LASSO (ALASSO) can reduce the model bias and prediction MSE by adding model variance. In an application of ridge regression, the LASSO and ALASSO to a simulated example based on results for 9,723 SNPs and 3,226 individuals, the best model selected was with the LASSO when *r^2^* was used as a measure. However, when model selection was based on test MSE and coefficient of determination *R^2^* the ALASSO proved to be the best method. Hence, use of *r^2^* may lead to selection of the wrong model and therefore also nonoptimal ranking of phenotype predictions and genomic breeding values. Instead, we propose use of the test MSE for model selection and *R^2^* as a standardized measure of the accuracy.

## Introduction

At the heart of classical quantitative genetics is linear model theory (Lynch and Walsh, 1998). Statistical inference in linear models mostly falls within the ordinary least squares (OLS) and maximum likelihood (ML) frameworks (Casella and Berger, 2002). The recent transition from pedigree-based classical quantitative genetics to prediction based on genome-wide markers involves some steps where the characteristics of the data complicate statistical inference and may have profound effects on model selection.

One of the most important factors is the number of markers *p* in relation to the number of individuals *n*. If *p* < < *n*, we can set up the linear model *y* = *X β* + *e* where each individual genotype score (0,1, or 2) is collected in a matrix *X* (standardized over columns to have mean equal to zero and variance equal to one) and the corresponding phenotypes in a vector *y* (centered to have a mean of zero), and then use standard OLS to obtain unbiased solutions to the regression coefficients of the genetic markers, i.e., *β_OLS_* = *(X^T^* *X)^-1^* *y*. Note that this is also the solution to the ML function $\hat{\beta }=\arg$ max *p(y* | *X, β)*. It is straightforward to incorporate dominance and epistasis into *X* using indicator variables. The predicted phenotypes are calculated as $\hat{y}=X\;\hat{\beta }$ and the residuals as *e* = *y* - *ŷ*. Based on the residuals, it is possible to calculate the residual sum of squares RSS = *e^T^* *e*, the OLS error variance $\sigma_{e}^{2}=\mathrm{RSS}/(n-p)$, and the mean squared error:

(1) $$\mathrm{MSE}=(1/n)\;\sum_{i=1}^{n}\;{\left(y_{i}-{\hat{y}}_{i}\right)}^{2}=\mathrm{RSS}/n.$$

We can also obtain the variances (diagonal terms) and covariances (off-diagonal terms) of the regression coefficients as $\mathrm{COV}[\hat{\beta }]=\sigma_{e}^{2}{\left(X^{T}X\right)}^{-1}$ (Ravishanker and Dey, 2002). However, for estimation of the genomic variance $\sigma_{g}^{2}$ and the genomic heritability $h_{g}^{2}=\sigma_{g}^{2}/(\sigma_{g}^{2}+\sigma_{e}^{2})$ it is necessary to use some random effects model where the covariance structure is based on the outer product *X X^T^* instead of the inner product *X^T^* *X* (Morota and Gianola, 2014; de los Campos et al., 2015). When *p* < < *n*, OLS will give unbiased estimates of the genomic parameters with low variance. However, if *n* is not much larger than *p*, there can be considerable variability in the OLS fit, resulting in overfitting with very small, or even zero error variance, and consequently incorrect predictions of future observations. Hence, it is advisable to cast OLS into a supervized statistical learning framework where the data are split into training and test sets, and MSE is evaluated on the test set (Hastie et al., 2009).

## Regularization

Although the number of genotyped individuals is generally increasing, the experimental setting in genomic prediction is often that *p* > *n* or even *p* > > *n*. This is an example of a high-dimensional statistical problem which leads to certain challenges (Johnstone and Titterington, 2009; Fan et al., 2014). Standard OLS is not applicable in this situation, because *X^T^* *X* is singular (i.e., does not have an inverse) and the parameters in the regression model cannot be uniquely estimated. One approach to overcome the singularity problem is to use regularization (also known as penalization). An early example of this is ridge regression (RR) (Hoerl and Kennard, 1970), in which the regression coefficient is estimated using ${\hat{\beta }}_{RR}={\left(X^{T}\;X+\lambda \;I_{p}\right)}^{-1}X^{T}y$ , where *I_p_* is an identity matrix and λ is a positive penalty parameter that needs to be tuned using training and test data. Note that genomic best unbiased linear prediction (GBLUP) is a form of random effects RR, where $\lambda =\sigma_{e}^{2}/\sigma_{g}^{2}$ and the genomic relationship matrix *G* is calculated based on *XX^T^* (Goddard, 2009; Morota and Gianola, 2014). There is also a Bayesian rationale for RR where the regression coefficients follows a normal prior, $\beta ∼N(0,(\sigma_{e}^{2}/\lambda )I)$. The RR estimator has some interesting properties. Firstly, both the expectation $E[{\hat{\beta }}_{RR}]$ and the variance $\mathrm{VAR}[{\hat{\beta }}_{RR}]$ tend towards zero when λ goes to infinity. Secondly, compared with OLS estimates, $E[{\hat{\beta }}_{RR}]$ is biased, and the variance of the OLS estimator $\mathrm{VAR}[{\hat{\beta }}_{OLS}]$ is always larger than $\mathrm{VAR}[{\hat{\beta }}_{RR}]$ when λ > 0 (van Wieringen, 2018).

Another interesting feature of RR appears when considering the MSE. In general, for any estimator of a parameter *θ*, the mean squared test error can be decomposed following $\mathrm{MSE}[\hat{\theta }]=\mathrm{VAR}[\hat{\theta }]\;+\;\mathrm{BIAS}{\left[\hat{\theta }\right]}^{2}$ (Hastie et al., 2009). The bias–variance decomposition is a way of analyzing the expected test error of a learning algorithm with respect to a particular problem. In order to minimize the test error, a model that simultaneously achieves low variance and low bias needs to be selected. The variance refers to the amount by which *θ* would change if it were estimated using other training datasets. Ideally, the estimate of *θ* should vary as little as possible. Bias represents the error that is the result of approximating a complex problem with a simpler model. Generally, more flexible methods result in less bias, but also lead to higher variance. Hence, there is a bias–variance trade-off that needs to be optimized using the test data. For data with an orthonormal design matrix, i.e., *X^T^* *X = I_p_* *= (X^T^* *X)^-1^* and *n = p*, it can be mathematically shown that there is a value of λ > 0 where$\mathrm{MSE}[{\hat{\beta }}_{RR}]<\mathrm{MSE}[{\hat{\beta }}_{OLS}]$ (Theobald, 1974; Farebrother, 1976).

RR can be written as an optimization problem $\min\{|y-X\beta {|}_{2}^{2}+\lambda |\beta {|}_{2}^{2}\}$, where $||\cdot |{|}_{2}$ denotes the Euclidean *ℓ*_2_-norm. The first term is the loss function and the second term the penalty. By changing the penalty into an *ℓ*_1_-norm, we end up with $\min\{|y-X\beta {|}_{2}^{2}+\lambda |\beta {|}_{1}\}$ which is also known as the LASSO (Tibshirani, 1996). In contrast to RR, the LASSO sets regression coefficients to zero and therefore performs variable selection. In general, the LASSO will perform better than RR when a relatively small number of predictors (markers) have relatively large effects on the response (phenotype). Compared with OLS, the LASSO and also RR can yield a reduction in variance at the expense of some increase in bias, and consequently generate lower MSE and better prediction accuracy (Hastie et al., 2009). Unfortunately, minimization of the LASSO problem does not provide an estimate of the error variance, because it depends on a complex relationship between the signal-to-noise ratio (i.e., the heritability) and the sparsity pattern (i.e., number of QTLs in relation to number of markers). In general, it is notoriously difficult to obtain proper error variance estimates with regularization methods in the *p* > *n* situation, because of the biased estimates and the difficulty in calculating correct degrees of freedom (Reid et al., 2016). The LASSO has been extended in many directions (Vidaurre et al., 2013; Hastie et al., 2015). Among the most interesting variants is the adaptive LASSO (ALASSO), where a pre-calculated vector *w* is used to weight the coefficients differently in the penalty, i.e., $\min\{|y-X\beta |{|}_{2}^{2}+\lambda ||w\beta |{|}_{1}\}$ (Zou, 2006). The weights can be calculated as the absolute values of marginal covariances between the markers and the phenotype. The bias introduced by the shrinkage of $\hat{\beta }$ in RR and LASSO is reduced in ALASSO at the expense of an increase in variance (Giraud, 2015). The LASSO and ALASSO have shown competitive prediction performance compared with a range of other methods in comparative genomic prediction studies (Li and Sillanpää, 2012; Momen et al., 2018).

## Model Selection

In order to determine the best model, it is important to find a good measure of the lowest test error, because the training error will decrease when more variables or parameters are added to the model. There are a number of approaches (e.g., Mallows’ *C_P_*, AIC and BIC) that attempt to correct the training RSS for model size. However, their use as model selection criteria in regularized models with *p* > *n* data is questionable, since they rely on asymptotic theory, for example that it is possible to obtain correct degrees of freedom and unbiased error variance estimates. In an application of RR to genomic marker data, Whittaker et al. (2000) suggest optimizing *λ* by minimizing $\mathrm{MSE}[{\hat{\beta }}_{RR}]=\mathrm{RSS}-n\sigma_{e}^{2}=\sigma_{e}^{2}tr[X^{T}X{\left(X^{T}X+\lambda I\right)}^{-1}]$, which is a variant of Mallows’ *C_P_*.

An alternative approach is to use cross-validation (CV). There are several variants of CV, but the general idea is to average MSE over some sets of hold-out test data (Hastie et al., 2009). In quantitative genetics, it is common to use the Pearson correlation coefficient, *r*, as a model selection criterion, both with and without CV (González-Recio et al., 2014). Daetwyler et al. (2008) suggest to use the expected predictive correlation accuracy:

(2) $$r^{2}={\left(\mathrm{COV}[y,\hat{y}]\right)}^{2}/(\mathrm{VAR}[y]\mathrm{VAR}[\hat{y}])$$

for model evaluation in genome-enabled prediction. The use of *r*^2^ for model comparison has been questioned, see for example Gianola and Schön (2016). Based on the regularization theory above, it is evident that there are potential problems with *r^2^* because VAR[*y*] will be unaffected, whereas VAR[ŷ] will be heavily influenced by the type of model and level of regularization.

It is also possible to assess the goodness of fit of the models using the coefficient of determination *R^2^*. Kvålseth (1985) identifies eight different variants of this statistic and compares them for different types of models. For linear OLS regression models with an intercept term, the problem seems to be of a minor nature, since the majority of the *R^2^* statistics are equivalent. However, for other types of models, such as linear models without intercepts or nonlinear models, the various *R^2^* statistics generally yield different values. Although not examined by Kvålseth (1985), the same problem applies to regularized models. Kvålseth (1985) concludes that the best coefficient to use is:

(3) $$R^{2}=1-\frac{\sum_{i=1}^{n}{\left(y_{i}-{\hat{y}}_{i}\right)}^{2}}{\sum_{i=1}^{n}{\left(y_{i}-\bar{y}\right)}^{2}}$$

## Illustration of the Problem With *r*^2^

In a recent publication (Waldmann et al., 2019), we presented a novel automatic adaptive LASSO (AUTALASSO) based on the alternating direction method of multipliers (ADMM) optimization algorithm. We also compared the ALASSO, LASSO, and RR on a simulated dataset using the glmnet software (Friedman et al., 2010). The original simulated data stem from the QTLMAS2010 workshop (Szydłowski and Paczynska, 2011). The total number of individuals is 3,226, structured in a pedigree with five generations. The continuous quantitative trait was created from 37 QTLs, including nine controlled major genes and 28 random minor genes. The controlled QTLs included two pairs of epistatic genes with no individual effects, three maternally imprinted genes, and two additive major genes. The random genes were chosen among the simulated SNPs and their effects were sampled from a truncated normal distribution. In addition to these original data, one dominance locus, one over-dominance and one under-dominance loci were created and added to the phenotype (Waldmann et al., 2019). The narrow sense heritability was equal to 0.45. MAF cleaning was performed at the 0.01 level, resulting in a final sample of 9,723 SNPs. Data from individual 1 to 2,326 were used as training data and data from individual 2,327 to 3,226 as test (or validation) data. The regularization path in glmnet was run over 100 different *λ*-values to estimate the smallest test MSE and largest test *r^2^* and *R^2^*.

In our previous paper (Waldmann et al., 2019), we estimated only MSE and *r^2^* and therefore add *R^2^* here. Application of the ALASSO, LASSO, and RR resulted in a test MSE of 64.52, 65.73, and 83.07, respectively. Hence, based on the MSE, it is clear that the ALASSO is the best model. The ALASSO is also favored in terms of *R^2^*, which yields the results 0.449, 0.439, and 0.291, respectively. However, based on *r^2^*, the LASSO is the best model, with an estimate of 0.460, compared with ALASSO and RR estimates of 0.455 and 0.300, respectively. Decomposing *r^2^* into its parts reveals that the test VAR[*y*] is the same (117.2) for all three methods. However, VAR[ŷ] differs between the models, increasing from 29.54 for RR to 36.41 for the LASSO and 48.17 for the ALASSO. The COV[*y*,ŷ] also follows this pattern, but the proportions to VAR[ŷ] differ. These results are summarized in **Table 1**. Introduction of the weight factor in the ALASSO increases model complexity, which results in decreased model bias at the expense of increased variance. Most importantly, however, the test MSE is reduced. This is an example of the bias-variance trade-off that is fundamental in statistical learning, where *r^2^* can provide estimates that may result in erroneous model decisions.

**Table 1:** Mean squared error (MSE), predictive correlation accuracy (*r^2^*), coefficient of determination (*R^2^*), covariance between test phenotypes and predicted test phenotypes (COV[y, ŷ]), and variance of predicted test phenotypes (VAR[ŷ]) for ridge regression (RR), LASSO and adaptive LASSO (ALASSO), evaluated on the simulated QTLMAS2010 data.

Method	MSE	*r^2^*	*R^2^*	COV[ *y*,ŷ]	VAR[ŷ]
RR	83.07	0.300	0.291	32.22	29.54
LASSO	65.73	0.460	0.439	44.30	36.41
ALASSO	64.52	0.455	0.449	50.68	48.17

Ranking of individuals in terms of breeding values and predicted phenotypes is important in breeding. The order of the 10 best individuals differs not only between the RR, LASSO and ALASSO, but also within each model when min MSE and max *r^2^* are used for determination of the best model (**Table 2**). How regularization and the variable selection properties of the LASSO and ALASSO affects the statistical properties of rank correlation measures (e.g. Spearman’s and Kendall’s rank correlation coefficients) is unclear because of the bias-variance trade-off and needs to be further investigated. For example, a rank correlation measure can be high even if the model is highly biased and therefore the rank statistic may work in the opposite direction of the MSE loss function which will lead to optimization conflicts. Hence, it would be necessary to use a model with a rank-based loss function.

**Table 2:** Ranking of the 10 best individuals from the simulated QTLMAS2010 data based on *ŷ* for RR, LASSO and ALASSO using min MSE and max predictive correlation accuarcy (*r^2^*) as model selection measures.

					Rank					
Metdod/selection statistic	1	2	3	4	5	6	7	8	9	10
RR/min[MSE]	2,586	2,772	2,977	3,050	3,195	3,056	2,756	2,738	2,821	3,184
RR/max[*r^2^*]	2,586	2,772	3,195	2,977	3,050	3,184	2,589	2,821	2,756	2,738
LASSO/min[MSE]	2,967	2,820	2,586	2,809	3,050	2,977	3,195	2,582	2,688	2,765
LASSO/max[*r^2^*]	2,967	2,820	2,809	2,688	2,582	2,586	3,195	3,050	2,977	2,972
ALASSO/min[MSE]	2,820	2,582	2,586	2,809	3,050	2,832	3,195	3,006	2,589	2,817
ALASSO/max[*r^2^*]	2,820	2,582	2,809	2,586	3,050	3,195	2,832	3,006	2,817	2,972

## Data Availability Statement

The simulated dataset QTLMAS2010ny012.zip can be found in https://github.com/patwa67/AUTALASSO.

## Author Contributions

The author wrote, read and approved the final version of the manuscript.

## Funding

Financial support was provided by the Beijer Laboratory for Animal Science, SLU, Uppsala.

## Conflict of Interest

The author declares that the research was conducted in the absence of any commercial or financial relationships that could be construed as a potential conflict of interest.
